# Erythrosine-induced hepatic biomarkers and histopathological changes in male rats: protective effects of lycopene and anthocyanin

**DOI:** 10.3389/fphar.2025.1718792

**Published:** 2026-01-08

**Authors:** Safa H. Qahl, Fatimah A. Alqahtani, Haleema Al-Nahari, Fawzyah A. Alghamdi, Fatimah H. Khouja, Amna H. Khouja

**Affiliations:** 1 Department of Biological Sciences, College of Science, University of Jeddah, Jeddah, Saudi Arabia; 2 Department of Biology, College of Science, University of King Khalid University, Abha, Saudi Arabia; 3 King Saud bin Abdulaziz University for Health Sciences, Jeddah, Saudi Arabia

**Keywords:** anthocyanin, hepatic function, lycopene, erythrosine, protective effects, rats, synthetic dyes

## Abstract

This study aimed to explore and reassess the safety and efficacy of the synthetic food dye erythrosine (ERY) with respect to the hepatic biomarkers and histological changes in male adult rats as well as possibly alleviate the effects of ERY through administration of lycopene (LYC) and anthocyanin (ANC). Sixty adult male rats were randomly distributed into six experimental groups as follows: control, LYC (5 mg/kg), ANC (200 mg/kg), ERY (20 mg/kg), ERY + LYC, and ERY + ANC. After 3 and 6 weeks of treatment, ERY (20 mg/kg) produced marked biochemical and hepatic injuries. ERY significantly reduced the total protein (from 6.93 g/dL in control to 5.33 g/dL) and albumin (from 2.65 to 1.77 g/dL, *p* < 0.050), whereas LYC and ANC co-administration improved these values compared to ERY alone (*p* < 0.050). ERY was also found to elevate the total cholesterol, triglycerides (TG), and low-density lipoprotein (LDL) values relative to the control, whereas both LYC and ANC lowered LDL and TG levels compared to the ERY-treated rats. The liver injury markers were strongly increased by ERY, with alanine transaminase (ALT) increasing from 14.00 to 23.95 U/L, aspartate transaminase (AST) increasing from 41.54 to 52.90 U/L, and gamma-glutamyl transferase (GGT) increasing from 6.24 to 8.64 U/L. Cotreatment with LYC or ANC was seen to significantly reduce ALT, AST, and GGT (*p* < 0.050). The total bilirubin increased significantly in the ERY group but was restored to near-control levels with both LYC and ANC. Histologically, ERY produced moderate-to-severe focal hepatic degeneration, including hepatocyte cytoplasmic vacuolation (score 2–3), nuclear chromatin clumping (score 2), focal necrosis (score 2), and leukocytic infiltration (score 2–3); LYC and ANC markedly ameliorated these lesions, reducing the pathological scores to 0–1, while restoring the hepatic cords, sinusoids, and nuclear morphology to near-normal or normal appearance by week 6. Collectively, ERY impacted the hepatic chemistry and functionality with considerable effect on the histological appearance of the hepatic tissues in rats, while ANC and LYC resourcefully attenuated these adverse effects.

## Introduction

Food preservatives are extensively found in numerous processed food products in the human environment. Food preservation is conventionally focused on three areas, namely, protecting the appearance of the food, prolonging the storage life of the food, and maintaining the nutritional activities of the food (Lindsay, 2007). The additional uptake of food additives or colorants upon food consumption has increased progressively and has become a significant problem necessitating attention. Chemical food additives have been shown to exhibit many changes in the defense system and immunity of the body, even leading to damage to some tissues and organs (Silva and Lidon, 2016; Sambu et al., 2022). Erythrosine (ERY) is one of the most commonly used synthetic food dyes in processed food products like snacks, beverages, and condiments as well as cosmetics. For a long time, ERY had been widely considered as a safe dye; however, some reports had invited reassessment of this consideration, especially in terms of its effects on thyroid function. In this sense, the Scientific Panel on Food Additives and Nutrient Sources Added to Food had reevaluated the safety of ERY when used as a food additive (Additives and Food, 2011). According to studies on animal models, the destructive impacts instigated by excessive use of ERY are hyperactivity, lesions, tumors, various allergic reactions, mutagenicity, genotoxicity (Mpountoukas et al., 2010), and impaired testicular function leading to orchitis (Wopara et al., 2021b). Research shows that ERY added as a colorant to processed foods may be toxic to the bodily organs (Gupta et al., 2019; Iheanyichukwu et al., 2021), and some of its implications in these tissues include leucopenia, anemia, renal dysfunction, hematological alterations, cardiovascular defects, and gastrointestinal disorders as well as interference with enzyme activities and functions.

ERY has been confirmed to be toxic to fish embryos (Gupta et al., 2019) and was also shown to increase the risk of neural tube defects in early-stage chicken embryos (Ovalioglu et al., 2020). Additionally, Marwa et al. (2019) found that ERY induced neurotransmitter imbalance and diminished the antioxidant balance in rats. This may be related to reduced antioxidative biomarkers as well as increased endogenous neurotoxins (Marwa et al., 2019) and oxidative/inflammatory pathways (Iheanyichukwu et al., 2021). Moreover, ERY was shown to promote myocardial remodeling in rats (Saqib and Maurya, 2019). The damaging effects of ERY on neurons (Marwa et al., 2019; Iheanyichukwu et al., 2021) and the kidney (Wopara et al., 2021a) have also been reported. However, further exploration of its effects on as well as mitigation strategies against changes to the hepatic chemistry and histology is still needed. The liver is a central part of many biological pathways involving several organs in the body. It is also one of the delicate organs in the body that is susceptible to contagions and impairment. Liver chemistry is often used to monitor the health of living organisms. The main function of the liver is sustaining homeostasis by eliminating toxins and harmful substances (Syrov et al., 2023).

Modifying dietary intake containing food additives could change the hepatic chemistry and is concerned with the safety or lethality of these compounds. Over the last decade, some reports have requested scientists to reassess the safety of food colorants like ERY based on experimental studies in rats (Additives and Food, 2011). Natural food additives comprising bioactive components and natural colorants have recently garnered substantial attention in the food industry (Nabi et al., 2023). Food consumers prefer the use of natural pigments in drinks and foods owing to their safety margins, given the destructive and unwanted side effects of synthetic pigments. Lycopene (LYC) is a carotenoid with a characteristic red color attributable to its conjugated polyene structure. LYC has been shown to have antioxidant and hepatoprotective effects (Abdel-Rahman et al., 2018; Syrov et al., 2023) against various diseases (Ibrahim et al., 2022). Another natural food pigment is anthocyanin (ANC), which is a type of plant phenolic pigment and dietary compound with potential health benefits and roles against human diseases (Tang et al., 2023); it has been shown to exhibit anti-inflammatory, antioxidant, neuroprotective, chemotherapeutic, cardioprotective, and hepatoprotective activities. The combination of LYC and ANC may confer enhanced hepatoprotective effects via complementary antioxidant and anti-inflammatory mechanisms. As a carotenoid, lycopene is a powerful scavenger of reactive oxygen species (ROS) that downregulates proinflammatory signaling (e.g., via AMPK/mTOR/NLRP3) and protects against cellular oxidative damage (Tan et al., 2024). As a class of flavonoid polyphenols, anthocyanins show hepatoprotective activity by directly scavenging free radicals, inducing nuclear translocation of Nrf2, upregulating downstream antioxidant enzymes like HO-1, and suppressing proinflammatory cytokines like TNFα (Mohammed and Khan, 2022). Moreover, anthocyanins are known to modulate metabolic-regulatory transcription factors: they influence the SIRT1/FOXO1 axis that supports both antioxidant defenses and anti-inflammatory signaling (Sabir et al., 2022). Lycopene primarily preserves the membrane lipids and lowers oxidative stress, whereas anthocyanin affects cell signaling and inflammatory gene expression more potently. When combined, LYC and ANC may work in concert. Compared to either compound alone, the combination may improve hepatocellular resilience, restore the biochemical markers, and preserve the liver histology. Therefore, assessing the combined hepatoprotective capabilities of these compounds makes biological sense and may have significant translational implications.

Given the biological features and health benefits of ANC and LYC to humans, we hypothesized that both natural pigments could afford hepatoprotective effects against ERY-instigated hepatic damage in a rat model. Thus, the present study was designed to deeply explore the actions of these compounds as well as reassess the potential mechanisms underlying the damage caused by ERY to the hepatic chemistry and histology; in particular, we considered the protective effects of the LYC and ANC molecules against ERY-induced damage.

## Materials and methods

### Ethical declaration

The current research was implemented under the supervision of the Animal Use in Research Committee at the University of Jeddah, Saudi Arabia, in accordance with the ethical recommendations of the National Institutes of Health (NIH) for the care and usage of laboratory animals in scientific studies. Moreover, the experiments were implemented as per the ARRIVE guidelines (Du Sert et al., 2020). The animal study was approved by the Institutional Ethical Committee at the Department of Biology, College of Science, University of Jeddah, Saudi Arabia (JU-20-2023) and was conducted in accordance with all local legislation and institutional requirements.

### Chemical reagents

**FIGURE 1:**
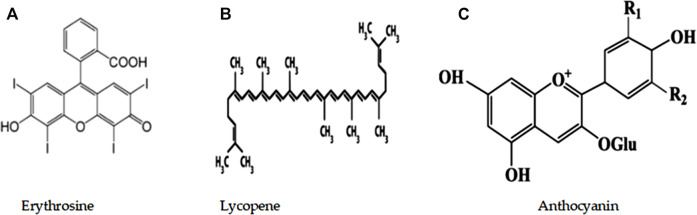
Chemical structural formulas of the materials investigated in this study: **(A)** erythrosine; **(B)** lycopene; **(C)** anthocyanin.

The compounds LYC, ANC, and ERY were procured from Sigma (St. Louis, MO, United States) and used as is. The chemical structures of these molecules are shown in Figure 1.

### Animal model

Sprague–Dawley albino male rats (60 rats, 170 ± 0.20 g, 8 weeks old) were acquired from the Department of Biology, University of Jeddah, Jeddah, Saudi Arabia, and used in the animal studies after 1 week of acclimation. The animals were housed in a well-ventilated room in stainless-steel cages with free access to water and food; the room had a controlled temperature of 22 °C ± 2 °C, relative humidity of 50%–60%, and was maintained under 12-h light/dark cycles. For 6 weeks, the animals were weighed and arbitrarily allotted into six groups (n = 10 per group) as follows: controls that were given distilled water orally, 5 mg of LYC per kilogram of bodyweight, 200 mg of ANC per kilogram of bodyweight, 20 mg of ERY per kilogram of bodyweight, and 20 mg of ERY (Iheanyichukwu et al., 2021) along with 5 mg of either LYC (LYC + ERY) (El-Kazaz et al., 2025) or 200 mg of ANC (ANC + ERY) (Pang et al., 2024) per kilogram of bodyweight. The doses of LYC (5 mg/kg) and ANC (200 mg/kg) were selected based on prior studies that demonstrated these concentrations to effectively exert antioxidant and protective effects in rat models without inducing toxicity. LYC at 5 mg/kg has been shown to significantly reduce oxidative stress as well as protect against liver and kidney damage through its strong free-radical-scavenging activity (Jiang et al., 2016). Similarly, ANC administered at 200 mg/kg was shown to improve antioxidant enzyme levels and protect against chemically induced oxidative damage in rats (Yasser and Sabour, 2023). These dosages were therefore selected in our study for their proven efficacies and safety profiles in comparable experimental models. All treatments were administered orally via orogastric gavage between 9 a.m. and 10 a.m. on alternate days. Every week, the number of treatments applied was adapted and established according to the bodyweight variabilities of the rats. The animal welfare parameters, such as injury, pain, irregular behaviors, discomfort, mucous membrane color, distress, morbidity, breathing patterns, and mortality, were all carefully checked through experimentation.

### Sample collection

At 3 and 6 weeks (end point) of treatments, the animals were fasted for 12 h before sample collection. All rats were balanced and anesthetized via an intramuscular injection of xylazine (5 mg/kg) and ketamine hydrochloride (50 mg/kg). The blood samples were collected from the medial canthus into sterilized tubes without anticoagulants. Then, these tubes were left at room temperature for 30 min to set the serum before centrifugation at 322×*g* for 20 min. The serum was carefully removed from each sample and deposited at −18 °C for further hormonal investigations. The rats were then euthanized by cervical dislocation (Aguwa et al., 2020); during the subsequent autopsy, the hepatic tissues of the rats were detached and immediately transferred for fixation with a buffered formalin solution (10%) for further histopathological investigations.

### Serum biochemistry assessments

The hepatic chemistry parameters, including total protein (TP), albumin (Alb), globulin (Glo), albumin/globulin ratio, total cholesterol (TC), low-density lipoprotein (LDL), high-density lipoprotein (HDL), total triglycerides (TG), aspartate transaminase (AST), alanine transaminase (ALT), gamma-glutamyl transferase (GGT), and bilirubin (Bil) levels, were assessed from the serum samples using enzyme-linked immunosorbent assay (ELISA) kits (specific to rats). All biochemical metrics were estimated using commercially available kits provided by BioDiagnostic Co. (Giza, Egypt). The hepatic enzymes AST and ALT were assessed according to the method reported by Yadav et al. (2022), whereas GGT was determined using the method reported by Gerussi et al. (2021). Bilirubin was further assessed using a colorimetric technique on a Hitachi 912 analyzer (Roche, Basel, Switzerland) following the protocol reported by Čepa et al. (2021). The TC and TG levels were evaluated using the techniques reported by Yadav et al. (2021) and McLelland et al. (2023), respectively. The HDL and LDL levels were assessed according to the method of Chary and Hedayati (2022). The TP and albumin levels were evaluated by the colorimetric method (Reinmuth-Selzle et al., 2022), while the globulin level was calculated from the TP and albumin values.

### Histopathological examination

The hepatic tissues were separated from each of the animals at 3 or 6 weeks of treatment, fixed and conserved in a formalin solution (10%), dehydrated in ascending scores of alcohol, cleared in xylene, and embedded and blocked in paraffin. A 3-µm-thick sample of each tissue was stained with eosin and hematoxylin (Alturkistani et al., 2015). Lastly, the slides were randomly evaluated by a specialized pathologist using a light microscope with blinding to the different study clusters.

### Statistical analysis and modeling

The data were edited using Microsoft Excel (Microsoft Corporation). The Levene and Shapiro–Wilk tests were used to check the normality and homogeneity of the variance (Demir, 2022). The general linear model of the statistical analysis system (Elliott and Woodward, 2023) was used to assess different treatments, time points, and their interactions. The mathematical model was set as follows:

$$Y_{\text{ijk}}=\mu +{\text{TRT}}_{i}+{\text{Tim}}_{j}+\text{TRT}*{\text{Tim}}_{k}+e_{\text{ijk}},$$

where Y is the studied gene; μ is the overall mean; TRT_i_ is the treatment effect (i = 1:5); Tim_j_ is the time effect (j = 1:2); TRT*Tim_k_ is the interaction effect between treatment and time; e_ijk_ is the error. In the event of significant effects, multiple comparisons among the means were carried out using Duncan’s multiple range test (Pagano et al., 2022). The figures were fitted using GraphPad Prism software 9.0 (GraphPad, United States), with the statistical significance set as *p* < 0.05. All results were expressed as means ± standard errors of the means (SEMs).

## Results

### Effects on protein fractions

**FIGURE 2:**
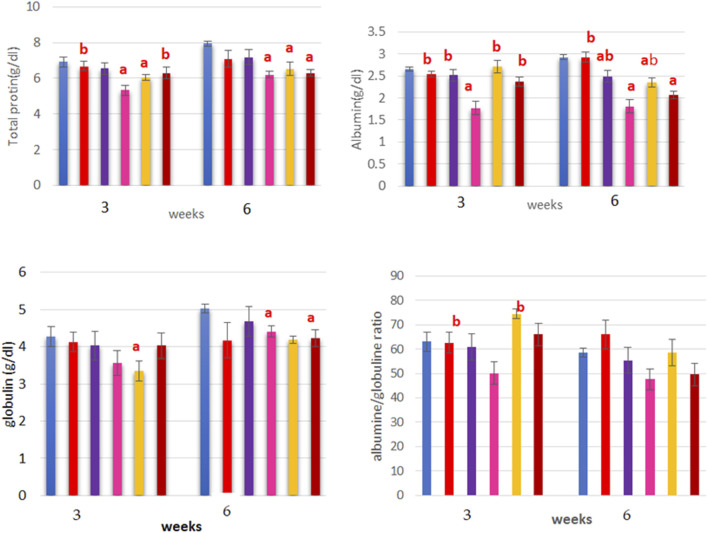
Treatment and time interaction (mean ± standard error of the mean (SEM)) effects based on oral administration of erythrosine (ERY) on the total protein (TP), albumin (Alb), globulin (Glo), and albumin/globulin (Alb/Glo) ratio in rats. ns, on-significant; *p < 0.05.

The data in Tables 1, 2 illustrate the effects of dietary LYC, ANC, ERY, and their combinations on the blood proteins, lipid profile, and liver function at 3 and 6 weeks, respectively. With respect to the blood proteins, the TP and albumin decreased significantly based on dietary treatment (*p* < 0.001) in the ERY-treated group than the control and other treatment groups (*p* < 0.05). Meanwhile, the lowest globulin levels were detected in the LYC and ERY groups. Regardless, the effects of treatment as well as interaction between treatment and time revealed that there were non-significant differences between the two time points for TP in all groups except the ERY group as there was a significant increase at 6 weeks post-treatment compared to that at 3 weeks (*p* < 0.05; Figure 2). Similarly, the concentrations of albumin and globulin showed significant increases (*p* < 0.05) at 6 weeks post-treatment compared to 3 weeks in all groups except the ANC and ERY groups for albumin as well as the LYC and ANC + ERY groups for globulin, which showed non-significant differences between the two sampling times (*p* > 0.05; Figure 2). The lowest albumin/globulin ratio was observed in the ERY-treated group, while the LYC, ANC, and ANC + ERY groups had similar values (Figure 2).

**TABLE 1:** Effects of treatment, sampling time, and their interactions on the blood hepatic chemistry of rats after 3 weeks of therapy.

Item	Treatment (T)*						SEM	*p*-value		
	CON	LYC	ANC	ERY	LYC + ERY	ANC + ERY		T	Time (Ti)	T × Ti
TP	6.93	6.68^b^	6.55	5.33^a^	6.04^a^	6.30^b^	0.173	<0.0001	0.0006	0.0028
Alb	2.65	2.54^b^	2.52^b^	1.77^a^	2.71^b^	2.37^b^	0.046	<0.0001	0.9313	<0.0001
Glo	4.28	4.14	4.03	3.56	3.34^a^	4.03	0.129	0.0015	<0.0001	0.0443
Alb/Glo	63.03	62.58^b^	60.95	50.14	87.02^b^	66	1.929	<0.0001	<0.0001	<0.0001
TC	94.58	90.4^a,b^	89.93^a,b^	109.25^a^	92.85	88.68^a,b^	0.580	<0.0001	<0.0001	0.0200
HDL	1.37	1.62^b^	1.64^a,b^	1.14^a^	1.59^b^	1.44^a,b^	0.015	<0.0001	<0.0001	<0.0001
LDL	0.26	0.30^a,b^	0.25^d^	0.35^a^	0.25^b^	0.29^a,b^	0.014	<0.0001	0.0160	<0.0001
TG	91.30	90.5^b^	90.35^b^	105.81^a^	98.93^a^	89.93^b^	1.011	<0.0001	<0.0001	<0.0001
Bil	1.57	1.63	1.32^b^	1.98	0.87^a,b^	0.88^a,b^	0.028	<0.0001	0.0181	<0.0001
ALT	14.00	12.52^a,b^	12.56^a,b^	23.95^a^	12.32^a,b^	20.79^a,b^	0.218	<0.0001	<0.0001	<0.0001
AST	41.54	38.56^a,b^	39.44^a,b^	52.90^a^	37.91^a,b^	33.06^a,b^	0.353	<0.0001	<0.0001	<0.0001
GGT	6.24	5.48^b^	4.17^a,b^	8.64^a^	4.88^a,b^	5.05^a,b^	0.096	<0.0001	0.4156	0.0124

TP, total protein; Alb, albumin; Glo, globulin; TC, total cholesterol; HDL, high-density lipoprotein; LDL, low-density lipoprotein; TG, triglycerides; Bil, bilirubin; ALT, alanine transaminase; AST, aspartate transaminase; GGT, gamma-glutamyl transferase; CON, control; LYC, lycopene; ANC, anthocyanin; ERY, erythrosine; SEM, standard error of the mean.

^a, b, c^ Means within a row without a common superscript letter differ at *p* < 0.05.

**TABLE 2:** Effects of treatment, sampling time, and their interactions on the blood hepatic chemistry of rats after 6 weeks of therapy.

Item	Treatment (T)*						SEM	*p*-value		
	CON	LYC	ANC	ERY	LYC + ERY	ANC + ERY		T	Time (Ti)	T × Ti
TP	7.95	7.10	7.17	6.21^a^	6.53^a^	6.30^a^	0.173	<0.0001	0.0006	0.0028
Alb	2.93	2.93^b^	2.49^a,b^	1.81^a^	2.35^a,b^	2.07^a^	0.046	<0.0001	0.9313	<0.0001
Glo	5.02	4.17	4.68	4.41^a^	4.18	4.32^a^	0.129	0.0015	<0.0001	0.0443
Alb/Glo	58.53	66	55.45	47.6	58.53	49.56	1.929	<0.0001	<0.0001	<0.0001
TC	75.61	69.67^a,b^	71.28^b^	85.02^a^	70.50^b^	68.45^a,b^	0.580	<0.0001	<0.0001	0.0200
HDL	1.58	1.62^b^	1.64^a,b^	1.14^a^	1.59^b^	1.44^a,b^	0.015	<0.0001	<0.0001	<0.0001
LDL	0.28	0.23^a,b^	0.23^a,b^	0.43^a^	0.3^b^	0.29^b^	0.014	<0.0001	0.0160	<0.0001
TG	100.79	91.03^a,b^	85.79^a,b^	148.83^a^	105.27^a,b^	110.67^a,b^	1.011	<0.0001	<0.0001	<0.0001
Bil	1.26	1.25^b^	1.16^b^	1.7^a^	1.21^b^	1.31^b^	0.028	<0.0001	0.0181	<0.0001
ALT	13.00	11.16^a,b^	12.19^b^	25.79^a^	14.02^b^	11.25^a,b^	0.218	<0.0001	<0.0001	<0.0001
AST	38.95	36.61^a,b^	39.71^a,b^	67.18^a^	35.87^a,b^	39.95^b^	0.353	<0.0001	<0.0001	<0.0001
GGT	6.50	5.23^a,b^	4.40^a,b^	8.95^a^	4.85^a,b^	4.66^a,b^	0.096	<0.0001	0.4156	0.0124

TP, total protein; Alb, albumin; Glo, globulin; TC, total cholesterol; HDL, high-density lipoprotein; LDL, low-density lipoprotein; TG, triglycerides; Bil, bilirubin; ALT, alanine transaminase; AST, aspartate transaminase; GGT, gamma-glutamyl transferase; CON, control; LYC, lycopene; ANC, anthocyanin; ERY, erythrosine; SEM, standard error of the mean.

^a, b, c^ Means within a row without a common superscript letter differ at *p* < 0.05.

#### Effects on lipid profile

**FIGURE 3:**
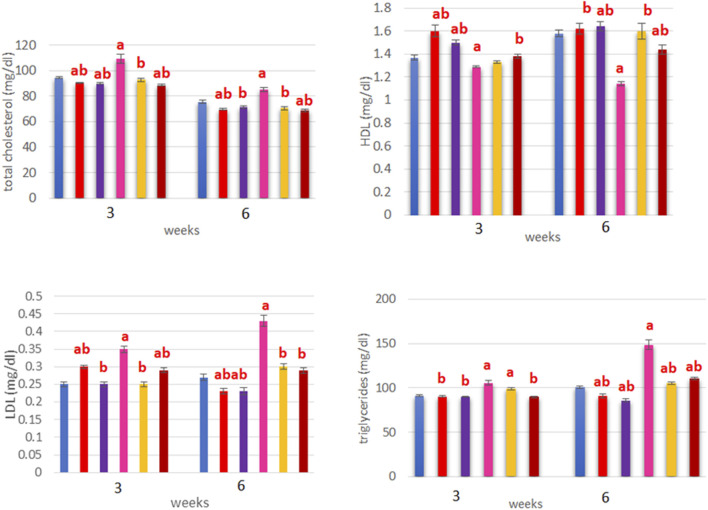
Treatment and time interaction (mean ± SEM) effects based on oral administration of ERY on the total cholesterol (TC), high-density lipoprotein (HDL), low-density lipoprotein (LDL), and triglycerides (TG) in rats. ns, non-significant; *p < 0.05.

In terms of the lipid profile, the TC concentrations were significantly low in all treated groups than the control, with the ERY-treated group showing a significant increase over the control and other groups (*p* < 0.05). Moreover, the ERY group showed the least HDL and highest LDL values compared to the control (*p* < 0.05). In terms of the TG, higher levels were observed in the ERY-treated and combined treatment groups than the control, LYC, and ANC groups. The results of interactions between treatment and time showed significant decreases in the TC cholesterol levels at 6 weeks post-treatment in all treatment groups than at 3 weeks (*p* < 0.05; Figure 3). The HDL levels were significantly higher at 6 weeks than at 3 weeks in the ANC and LYC + ERY groups (*p* < 0.05); however, HDL was significantly lower at 6 weeks than 3 weeks post-treatment in the ERY-treated group (*p* < 0.05; Figure 3). The LDL and TG values were both significantly higher at 6 weeks than 3 weeks post-treatment in the ERY and LYC + ERY groups (*p* < 0.05; Figure 3).

### Effects on liver enzymes

**FIGURE 4:**
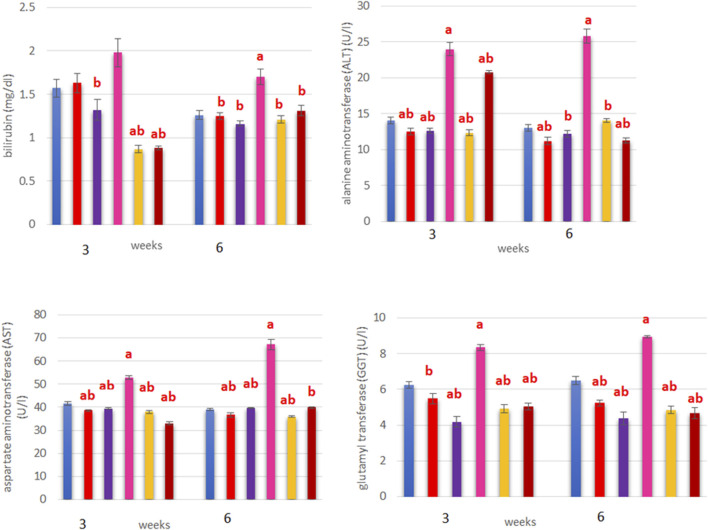
Treatment and time interaction (mean ± SEM) effects based on the oral administration of ERY on the total bilirubin (Bil), alanine aminotransferase (ALT), aspartate aminotransferase (AST), and gamma-glutamyl transferase (GGT) in rats. ns, non-significant; *p < 0.05.

In terms of liver function, the levels of total bilirubin as well as the enzymes ALT, AST, and GGT were significantly higher in the ERY-treated group than in the control and other groups (*p* < 0.05). The interactions between treatment and time indicated that the levels of Bil between the two time periods were significantly lower at 6 weeks post-treatment than at 3 weeks in the LYC, ANC, and ERY groups; however, Bil levels were significantly higher at 6 weeks than 3 weeks post-treatment in both the combination groups (*p* < 0.05; Figure 4). The activity of ALT was significantly higher at 3 weeks than 6 weeks post-treatment in the LYC and ANC + ERY groups and vice versa in the LYC + ERY group (*p* < 0.05; Figure 4). In terms of the AST and GGT levels, most of the treatment groups showed non-significant differences between the two time points, except ERY that showed a significant increase in AST and significant decrease in GGT levels at 3 weeks than at 6 weeks post-treatment (*p* < 0.05; Figure 4).

### Hepatic histological examinations

**FIGURE 5:**
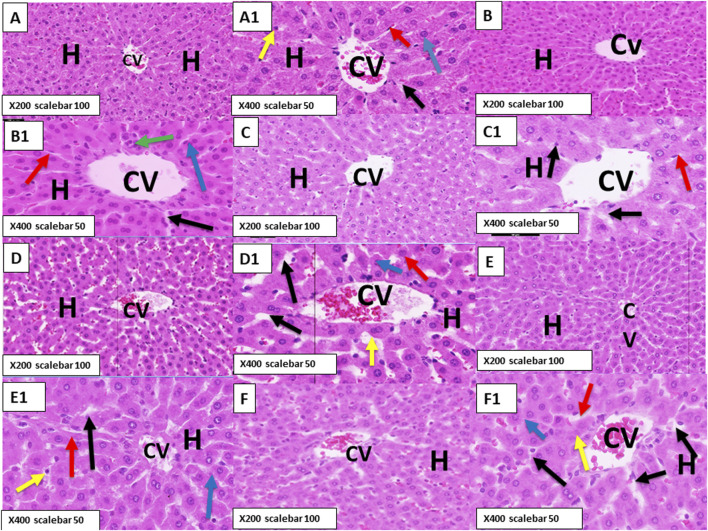
**(A,A1)** Photomicrograph of the control rat liver stained with hematoxylin and eosin showing normal hepatic parenchyma. The hepatocytes (H) radiating from the central vein (CV) have rounded vesicular nuclei (blue arrow) and are separated from each other by blood sinusoids (black arrow) lined with endothelial cells (red arrow) and von Kupffer cells (yellow arrow). **(B,B1)** Photomicrograph of rats treated with lycopene (LYC) for 3 weeks showing normal hepatocytes (H) with normal nuclei (blue arrow), normal blood sinusoids (black arrow) nearly similar to those of the control rats, and a few mononuclear cell infiltrations (green arrow). **(C,C1)** Photomicrograph of rats treated with anthocyanin (ANC) for 3 weeks showing no detectable changes compared to the control and LYC-treated groups. **(D,D1)** Photomicrograph of rats treated with ERY for 3 weeks showing dilatation and congestion in the CV and dilatation in the blood sinusoids (black arrows) between the hepatocytes (H). The hepatocytes show deeply stained cytoplasm and some deeply stained nuclei (blue arrow). **(E,E1)** Photomicrograph of rats treated with a combination of LYC and ERY for 3 weeks showing normal hepatocytes (H) with vesicular nuclei (blue arrow) and normal blood sinusoids (black arrow). **(F,F1)** Photomicrograph of rats treated with a combination of ANC and ERY for 3 weeks showing dilatation and congestion in the CV and dilatation in the blood sinusoids (black arrows) between the hepatocytes (H). The hepatocytes are normal with vesicular nuclei (blue arrow). (X200 scale bar: 100 µm; X400 scale bar: 50 µm).

**FIGURE 6:**
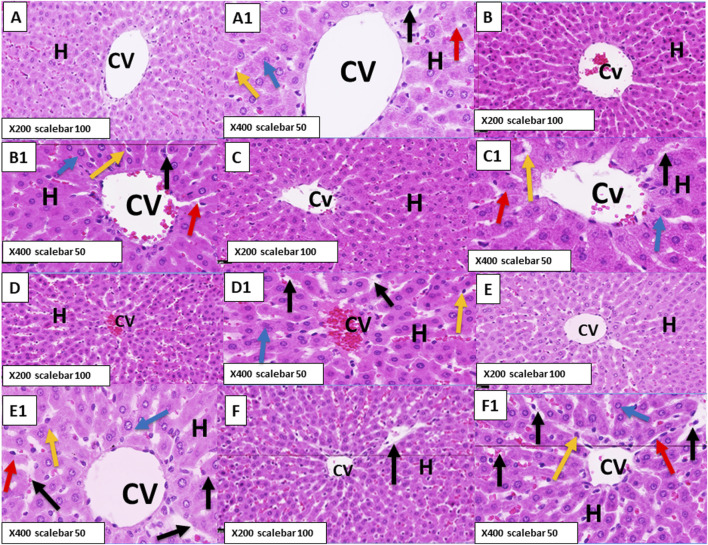
**(A,A1)** Photomicrograph of the control rat liver stained with hematoxylin and eosin showing normal hepatic parenchyma. The hepatocytes (H) radiating from the CV show rounded vesicular nuclei (blue arrow) and are separated from each other by blood sinusoids (black arrow) lined with endothelial cells (red arrow) and von Kupffer cells (yellow arrow). **(B,B1)** Photomicrograph of rats treated with LYC for 6 weeks showing normal hepatocytes (H) with normal nuclei (blue arrow), normal blood sinusoids (black arrow) that are nearly similar to those of the control group, and a few mononuclear cell infiltrations (green arrow). **(C,C1)** Photomicrograph of rats treated with ANC for 6 weeks showing no detectable changes compared to the control and LYC-treated groups. **(D,D1)** Photomicrograph of rats treated with ERY for 6 weeks showing congestion in the CV and dilatation in the blood sinusoids (black arrows) between the hepatocytes (H). The hepatocytes show deeply stained cytoplasm and some deeply stained nuclei (blue arrow). **(E,E1)** Photomicrograph of rats treated with a combination of LYC and ERY for 6 weeks showing normal structure of the liver and normal hepatocytes (H) with vesicular nuclei (blue arrow). **(F,F1)** Photomicrograph of rats treated with a combination of ANC and ERY for 6 weeks showing dilatation in the blood sinusoids (black arrow) between the hepatocytes (H). The hepatocytes are normal with vesicular nuclei (blue arrow). (X200 scale bar: 100 µm; X400 scale bar: 50 µm).

Examination of the hematoxylin-and-eosin-stained sections of the liver of healthy control rats showed normal hepatic architecture divided into ill-defined classic hepatic lobules. The classic lobules formed by cords of hepatocytes radiated outward from the central vein (CV) to the peripheries of the lobules. The portal triads or tracts were distributed at the corners of the lobules and surrounded by loose stromal connective tissue. The hepatocytes were polyhedral in shape, with strongly eosinophilic cytoplasm dotted with basophilic granules as well as distinct vesicular rounded nuclei and one or two prominent nucleoli. The hepatocytes were frequently binucleated. The CV was lined with flat endothelial cells, and hepatic blood sinusoids were observed between the hepatic cell cords lined with endothelial cells and von Kupffer cells containing ovoid nuclei (Figures 5A, 6A).

Rats treated with LYC for 3 and 6 weeks (Figures 5B, 6B) showed normal hepatocytes with normal nuclei and normal blood sinusoids that were nearly similar to those of the control group except for a few mononuclear cell infiltrations (green arrow). Our results revealed that treatment with ANC for 3 and 6 weeks (Figures 5C, 6C) showed no detectable changes compared to the control and LYC treatment groups.

Treatment with ERY for 3 and 6 weeks showed dilatation and congestion in the CV as well as dilatation in the blood sinusoids between the hepatocytes (Figures 5D, 6D); the hepatocytes showed deeply stained cytoplasm, with some showing deeply stained nuclei.

Rats treated for 3 and 6 weeks with a combination of LYC and ERY showed normal hepatocytes with vesicular nuclei and normal blood sinusoids that were nearly similar to those of the control group (Figures 5E, 6E). Rats treated for 3 and 6 weeks with a combination of ANC and ERY showed dilatation and congestion in the central vein as well as dilatation in the blood sinusoids between the hepatocytes; the hepatocytes were normal with vesicular nuclei (Figures 5F, 6F).

## Discussion

The liver is the primary region of xenobiotic metabolism and is extremely susceptible to harmful influences. In the present work, we observed notably diminished hepatic functions in rats that were orally administered ERY for 6 weeks, which resulted in significant increases in the lipid profile (TC and TG) as well as hepatic enzymes GGT, AST, and ALT than in the control animals. Moreover, the protein fractions were significantly decreased owing to ERY administration in the rats, which could be mitigated by the natural pigments LYC and ANC. These findings may be interrelated with the pathological changes in hepatic tissues prompted by ERY administration, including vacuolar degeneration of the hepatocytes with growth of pyknotic nuclei and Kupffer cells. The changes in the protein fractions reflect pathogens that may affect the host; in this work, the protein fractions in the ERY groups were significantly lower than in the other groups. In contrast, some authors reported that ERY administration significantly increased the release of TP in the hepatic or brain tissues of rats (Mekkawy et al., 2000; Chequer et al., 2012). Moreover, earlier studies have confirmed the mutagenic activity of ERY (Hamdy et al., 2000). In the present study, the insufficient production of globulins and albumins may reflect hepatic dysfunctionality. Albumin supports transportation of bilirubin, hormones, vitamins, metals, and drugs; accordingly, bilirubin accumulated in the serum of the ERY group owing to less albumin production. These results are in line with other reports that there is a distinct affinity for binding between bilirubin and albumin (Cahyana and Gordon, 2013; Zhang et al., 2018). Many of these glycosylated compounds also exhibited diminished affinity for binding with ANC (Zhang et al., 2018). We noted a significant decrease in the bilirubin level in the ANC group, which is in line with findings that ANC competitively inhibits the expression of bilirubin transporter families in the liver (Passamonti et al., 2002; Pelizzo et al., 2023). Our results show a significant decrease in bilirubin level in the LYC group along with decreased globulin and albumin concentrations; LYC’s capability to preserve these concentrations within typical boundaries demonstrates its curative abilities in alternative and complementary medicine (Eze et al., 2019). However, LYC and ANC may enhance the release and biosynthesis of albumin, thus reducing the transportation of bilirubin.

ERY has an important role in fat metabolism and binds fatty acids to keep them soluble in the plasma. Accordingly, we noted lipid accumulation in the sera of ERY rats in this work. ERY significantly increased the TG and TC levels in rats, which may be responsible for normal myocardial function (Saqib and Maurya, 2019). However, LYC modulated lipid metabolism by reducing oxidative stress and enhancing the beta oxidation of fatty acids in the liver, thus preventing lipid accumulation (Cao et al., 2021). The antiobesity impact of LYC has also been evidenced in earlier works (Fenni et al., 2017; Albrahim and Alonazi, 2021). Administration of ANC to high-fat-fed mice also showed numerous changes in obesity-associated indices, including improved β-cell functions as well as fasting blood glucose and leptin concentrations (Meydani and Hasan, 2010). In addition, ANC alleviated endotoxemia as well as decreased NOX4 and TNFα overexpression along with JNK phosphorylation, indicating that it could alleviate the inflammatory and pro-oxidant conditions stimulated by extra fat uptake (Cremonini et al., 2022). When the hepatocyte membrane is compromised, the cytosolic enzymes are released unconstrained into the bloodstream. Thus, the augmented GGT, AST, and ALT enzyme activities could help elucidate the damage to the hepatocytes, particularly in the ERY-treated group. Some results regarding these effects were described by Abd-Elhakim et al. (2023), who noted that some synthetic food additives significantly increased AST and ALT levels in rats; the ERY-exposed rats showed considerably increased GGT, AST, and ALT levels. The increases in these enzymes may be attributed to the cytotoxic influence of ERY, which can cause damage to the hepatocytes (Iheanyichukwu et al., 2021; Wopara et al., 2021b; Abd-Elhakim et al., 2023). In contrast, both natural pigments (LYC and ANC) significantly mitigated these increases in the liver enzymes. As documented previously, this confirms the hepatoprotective effects of LYC (Abdel-Rahman et al., 2018) and ANC (Tang et al., 2023). Recent studies have also revealed the hepatoprotective functions of LYC through its antioxidant, antiviral, and anti-inflammatory activities (Ibrahim et al., 2022; Syrov et al., 2023). GGT also plays a substantial role in metabolizing drugs and other hazardous elements in the hepatocytes.

The inhibitory effects of ERY on alkaline phosphatase (ALP) enzyme action may be associated with their interface with Zn as ALP is a Zn^2+^ metalloprotein (Abd-Elhakim et al., 2023). It has been reported that some synthetic food colorants may have inhibitory effects on ALP and its destructive processing in the hepatic cells at chronic levels, resulting in hepatic dysfunction (Abd-Elhakim et al., 2023). Another hypothesis suggested that synthetic food elements may induce higher oxidative stress, which could attach fatty acids to the cell membranes to instigate mitochondrial impairment and dysfunction (Yang et al., 2023) resulting in enzyme leakage.

For more insights into the liver chemistry, we investigated the histopathological architecture of the hepatic tissue in response to ERY administration. Based on the hematoxylin and eosin staining results, ERY induced increases in hepatocyte cytoplasm vacuolation, cytoplasmic basophily, clumping of nuclear chromatin, necrosis, and leukocyte probing of the walls of blood vessels to infiltrate the surrounding tissues.

These results can also be correlated with the pathological changes in the hepatocytes upon ERY administration based on the deeply stained cytoplasm and nuclei (pyknotic nuclei). Previously, Abd-Elhakim et al. (2023) found that some synthetic food colorants induced considerable changes in the vacuolar degeneration of hepatocytes, with pyknotic nuclei and Kupffer cell growth, in rats. Based on our present work, cotreatment with either LYC or ANC can successfully reestablish and recover near-control hepatic morphology through the hepatoprotective actions of LYC and ANC, as also clarified in previous works (Abdel-Rahman et al., 2018; Ibrahim et al., 2022; Syrov et al., 2023). Based on the histological architecture of the hepatic tissue in rats, ERY impacted the hepatic chemistry and functionality considerably while ANC and/or LYC could resourcefully attenuate these adverse effects.

## Conclusion

In this study, we show that 6 weeks of oral administration of food preservatives like ERY in a rat model substantially escalates hepatic enzyme leakage, serum lipid profile, protein fraction, and numerous histopathological disturbances in the hepatic tissues of male rats. Furthermore, natural food pigments like ANC and LYC exhibit protective effects on most hepatic functions and histopathological consequences; these pigments could mitigate the determinable effects of hepatic dysfunction induced by ERY in the rat model. Our findings affirm the significance of restricting the use of food preservatives and applying them only within the legal limits in industrial food products. Further investigations may be essential to shift awareness regarding the use of food preservatives as safe components, which would have consequences for more discreet usage of such additives. Additionally, rigorous reconnaissance assessments by public and food safety authorities are necessitated before applying such synthetic substances in food preparation and preservation.

## Data Availability

The raw data supporting the conclusions of this article will be made available by the authors, without undue reservation.

## References

[B1] Abd-Elhakim Y. M. Behairy A. Hashem M. M. M. Abo-El-Sooud K. El-Metwally A. E. Hassan B. A. (2023). Toll-like receptors and nuclear factor kappa B signaling pathway involvement in hepatorenal oxidative damage induced by some food preservatives in rats. Sci. Rep. 13 (1), 5938. 10.1038/s41598-023-32887-9 37045926 PMC10097866

[B2] Abdel-Rahman H. G. Abdelrazek H. M. A. Zeidan D. W. Mohamed R. M. Abdelazim A. M. (2018). Lycopene: hepatoprotective and antioxidant effects toward bisphenol A-Induced toxicity in female wistar rats. Oxid. Med. Cell Longev. 2018, 5167524. 10.1155/2018/5167524 30147835 PMC6083545

[B3] Additives E. P. o. F. Food N. S. a. t. (2011). Scientific opinion on the re-evaluation of erythrosine (E 127) as a food additive. EFSA J. 9 (1), 1854. 10.2903/j.efsa.2011.1854

[B4] Aguwa U. Eze C. Obinwa B. Okeke S. Onwuelingo S. Okonkwo D. (2020). Comparing the effect of methods of rat euthanasia on the brain of wistar rats: cervical dislocation, chloroform inhalation, diethyl ether inhalation and formalin inhalation. J. Adv. Med. Med. Res. 32 (17), 8–16. 10.9734/JAMMR/2020/v32i1730636

[B5] Albrahim T. Alonazi M. A. (2021). Lycopene corrects metabolic syndrome and liver injury induced by high fat diet in Obese rats through antioxidant, anti-inflammatory, antifibrotic pathways. Biomed. and Pharmacother. 141, 111831. 10.1016/j.biopha.2021.111831 34237596

[B6] Alturkistani H. A. Tashkandi F. M. Mohammedsaleh Z. M. (2015). Histological stains: a literature review and case study. Glob. Journal Health Science 8 (3), 72–79. 10.5539/gjhs.v8n3p72 26493433 PMC4804027

[B7] Cahyana Y. Gordon M. H. (2013). Interaction of anthocyanins with human serum albumin: influence of pH and chemical structure on binding. Food Chem. 141 (3), 2278–2285. 10.1016/j.foodchem.2013.05.026 23870958

[B8] Cao C. Sun S. Li J. Song C. Meng Q. Shi B. (2021). Lycopene modulates lipid metabolism in rats and their offspring under a high-fat diet. Food Funct. 12 (19), 8960–8975. 10.1039/d1fo01039e 34378595

[B9] Čepa A. Dejmková V. Lešetický L. Jelínek I. Smrček S. Štícha M. (2021). Physico-chemical characterization of bilirubin-10-sulfonate and comparison of its acid–base behavior with unconjugated bilirubin. Sci. Rep. 11 (1), 12896. 10.1038/s41598-021-92377-8 34145377 PMC8213708

[B10] Chary A. Hedayati M. (2022). Review of laboratory methods to determine HDL and LDL subclasses and their clinical importance. Rev. Cardiovasc. Med. 23 (4), 147. 10.31083/j.rcm2304147 39076233 PMC11273998

[B11] Chequer F. M. Venancio V. P. Bianchi M. L. Antunes L. M. (2012). Genotoxic and mutagenic effects of erythrosine B, a xanthene food dye, on HepG2 cells. Food Chem. Toxicol. 50 (10), 3447–3451. 10.1016/j.fct.2012.07.042 22847138

[B12] Cremonini E. Daveri E. Iglesias D. E. Kang J. Wang Z. Gray R. (2022). A randomized placebo-controlled cross-over study on the effects of anthocyanins on inflammatory and metabolic responses to a high-fat meal in healthy subjects. Redox Biol. 51, 102273. 10.1016/j.redox.2022.102273 35255426 PMC8902616

[B13] Demir S. (2022). Comparison of normality tests in terms of sample sizes under different skewness and Kurtosis coefficients. Int. J. Assess. Tools Educ. 9 (2), 397–409. 10.21449/ijate.1101295

[B14] Du Sert N. P. Ahluwalia A. Alam S. Avey M. T. Baker M. Browne W. J. (2020). Reporting animal research: explanation and elaboration for the ARRIVE guidelines 2.0. PLoS Biology 18 (7), e3000411. 10.1371/journal.pbio.3000411 32663221 PMC7360025

[B15] El-Kazaz S. E. Hafez M. H. Noreldin A. E. Khafaga A. F. (2025). Lycopene alleviates cognitive dysfunctions in an Alzheimer's disease rat model *via* suppressing the oxidative and neuroinflammatory signaling. Tissue Cell 96, 102975. 10.1016/j.tice.2025.102975 40378674

[B16] Elliott A. C. Woodward W. A. (2023). SAS essentials: mastering SAS for data analytics. 3rd edn. Hoboken, NJ: John Wiley & Sons.

[B17] Eze E. D. Afodun A. M. Kasolo J. Kasozi K. I. (2019). Lycopene improves on basic hematological and immunological parameters in diabetes mellitus. BMC Res. Notes 12 (1), 805. 10.1186/s13104-019-4841-8 31831054 PMC6909647

[B18] Fenni S. Hammou H. Astier J. Bonnet L. Karkeni E. Couturier C. (2017). Lycopene and tomato powder supplementation similarly inhibit high-fat diet induced obesity, inflammatory response, and associated metabolic disorders. Mol. Nutr. Food Res. 61 (9), 1601083. 10.1002/mnfr.201601083 28267248

[B19] Gerussi A. Bernasconi D. P. O'Donnell S. E. Lammers W. J. Van Buuren H. Hirschfield G. (2021). Measurement of gamma glutamyl transferase to determine risk of liver transplantation or death in patients with primary biliary cholangitis. Clin. Gastroenterol. Hepatol. 19 (8), 1688–1697 e1614. 10.1016/j.cgh.2020.08.006 32777554

[B20] Gupta R. Ranjan S. Yadav A. Verma B. Malhotra K. Madan M. (2019). Toxic effects of food colorants erythrosine and tartrazine on zebrafish embryo development. Curr. Res. Nutr. Food Sci. J. 7 (3), 876–885. 10.12944/CRNFSJ.7.3.26

[B21] Hamdy A. Mekkawy A. Massoud A. El-Zawahry M. (2000). Mutagenic effects of the food colour erythrosine in rats. Probl. Forensic Sci. 43, 184–191.

[B22] Ibrahim I. M. Althagafy H. S. Abd-Alhameed E. K. Al-Thubiani W. S. Hassanein E. H. M. (2022). Promising hepatoprotective effects of lycopene in different liver diseases. Life Sci. 310, 121131. 10.1016/j.lfs.2022.121131 36306869

[B23] Iheanyichukwu W. Adegoke A. O. Adebayo O. G. Emmanuel U. M. Egelege A. P. Gona J. T. (2021). Combine colorants of tartrazine and erythrosine induce kidney injury: involvement of TNF-alpha gene, caspase-9 and KIM-1 gene expression and kidney functions indices. Toxicol. Mech. Methods 31 (1), 67–72. 10.1080/15376516.2020.1828523 32981412

[B24] Jiang W. Guo M. H. Hai X. (2016). Hepatoprotective and antioxidant effects of lycopene on non-alcoholic fatty liver disease in rat. World J. Gastroenterol. 22 (46), 10180–10188. 10.3748/wjg.v22.i46.10180 28028366 PMC5155177

[B25] Lindsay R. C. (2007). Food additives *Fennema's food chemistry* . CRC Press, 701–762.

[B26] Marwa G. B. Magdy M. M. Maha M. K. Omayma H. (2019). Effect of synthetic coloring dye erythrosine (E127) in rats. J. Environ. Sci. 46 (1), 21–34. 10.21608/jes.2019.67965

[B27] McLelland G. L. Lopez-Osias M. Verzijl C. R. C. Ellenbroek B. D. Oliveira R. A. Boon N. J. (2023). Identification of an alternative triglyceride biosynthesis pathway. Nature 621 (7977), 171–178. 10.1038/s41586-023-06497-4 37648867 PMC10482677

[B28] Mekkawy H. Massoud A. El-Zawahry A. (2000). Mutagenic effects of the food color erythrosine in rats. Probl. Forensic Sci. 43, 184–191.

[B29] Meydani M. Hasan S. T. (2010). Dietary polyphenols and obesity. Nutrients 2 (7), 737–751. 10.3390/nu2070737 22254051 PMC3257683

[B30] Mohammed H. A. Khan R. A. (2022). Anthocyanins: traditional uses, structural and functional variations, approaches to increase yields and products' quality, hepatoprotection, liver longevity, and commercial products. Int. J. Mol. Sci. 23 (4), 2149. 10.3390/ijms23042149 35216263 PMC8875224

[B31] Mpountoukas P. Pantazaki A. Kostareli E. Christodoulou P. Kareli D. Poliliou S. (2010). Cytogenetic evaluation and DNA interaction studies of the food colorants amaranth, erythrosine and tartrazine. Food Chem. Toxicol. 48 (10), 2934–2944. 10.1016/j.fct.2010.07.030 20667460

[B32] Nabi B. G. Mukhtar K. Ahmed W. Manzoor M. F. Ranjha M. M. A. N. Kieliszek M. (2023). Natural pigments: anthocyanins, carotenoids, chlorophylls, and betalains as colorants in food products. Food Biosci. 52, 102403. 10.1016/j.fbio.2023.102403

[B33] Ovalioglu A. O. Ovalioglu T. C. Arslan S. Canaz G. Aydin A. E. Sar M. (2020). Effects of erythrosine on neural tube development in early chicken embryos. World Neurosurg. 134, e822–e825. 10.1016/j.wneu.2019.11.017 31715407

[B34] Pagano M. Gauvreau K. Mattie H. (2022). Principles of biostatistics. Chapman and Hall/CRC.

[B35] Pang Y. Men J. Li Y. Zhang J. Zhao L. Wang H. (2024). Blueberry anthocyanins regulate SIRT1/FoxO1 pathway to inhibit oxidative stress and reduce testicular tissue damage induced by microwave radiation in rats. J. Funct. Foods 122, 106523. 10.1016/j.jff.2024.106523

[B36] Passamonti S. Vrhovsek U. Mattivi F. (2002). The interaction of anthocyanins with bilitranslocase. Biochem. Biophys. Res. Commun. 296 (3), 631–636. 10.1016/s0006-291x(02)00927-0 12176028

[B37] Pelizzo P. Stebel M. Medic N. Sist P. Vanzo A. Anesi A. (2023). Cyanidin 3-glucoside targets a hepatic bilirubin transporter in rats. Biomed. Pharmacother. 157, 114044. 10.1016/j.biopha.2022.114044 36463829

[B38] Reinmuth-Selzle K. Tchipilov T. Backes A. T. Tscheuschner G. Tang K. Ziegler K. (2022). Determination of the protein content of complex samples by aromatic amino acid analysis, liquid chromatography-UV absorbance, and colorimetry. Anal. Bioanal. Chem. 414 (15), 4457–4470. 10.1007/s00216-022-03910-1 35320366 PMC9142416

[B39] Sabir U. Irfan H. M. Umer I. Niazi Z. R. Asjad H. M. M. (2022). Phytochemicals targeting NAFLD through modulating the dual function of forkhead box O1 (FOXO1) transcription factor signaling pathways. Naunyn Schmiedeb. Arch. Pharmacol. 395 (7), 741–755. 10.1007/s00210-022-02234-2 35357518

[B40] Sambu S. Hemaram U. Murugan R. Alsofi A. A. (2022). Toxicological and teratogenic effect of various food additives: an updated review. BioMed. Res. Int. 2022, 6829409. 10.1155/2024/9792751 35782077 PMC9249520

[B41] Saqib J. Maurya H. (2019). Biochemical and pharmacological estimation of erythrosine in myocardial remodeling among heart failure induced by doxorubicin in rats. Asian J. Pharm. Pharmacol. 5 (1), 43–48. 10.31024/ajpp.2019.5.1.5

[B42] Silva M. M. Lidon F. (2016). Food preservatives–An overview on applications and side effects. Emir. J. Food Agric. 28, 366–373. 10.9755/ejfa.2016-04-351

[B43] Syrov V. Gusakova S. Khushbaktova Z. Egamova F. Khidoyatova S. K. Sagdullaev S. S. (2023). Hepatoprotective efficacy of a new phytocomposition of essential phospholipids with glycyrrhizic acid, ecdysterone, and lycopene in experimental chronic hepatitis compared to phosphogliv. Pharm. Chem. J. 56 (11), 1433–1438. 10.1007/s11094-023-02811-6

[B44] Tan C. Chen J. Tu T. Chen L. Zou J. (2024). Lycopene inhibits pyroptosis of endothelial progenitor cells induced by ox-LDL through the AMPK/mTOR/NLRP3 pathway. Open Med. 19 (1), 20240973. 10.1515/med-2024-0973 38919547 PMC11197008

[B45] Tang C. Han J. Chen D. Zong S. Liu J. Kan J. (2023). Recent advances on the biological activities of purple sweet potato anthocyanins. Food Biosci. 53, 102670. 10.1016/j.fbio.2023.102670

[B46] Wopara I. Adebayo O. G. Umoren E. B. Aduema W. Iwueke A. V. Etim O. E. (2021a). Involvement of striatal oxido-inflammatory, nitrosative and decreased cholinergic activity in neurobehavioral alteration in adult rat model with oral co-exposure to erythrosine and tartrazine. Heliyon 7 (11), e08454. 10.1016/j.heliyon.2021.e08454 34888423 PMC8637136

[B47] Wopara I. Modo E. U. Mobisson S. K. Olusegun G. A. Umoren E. B. Orji B. O. (2021b). Synthetic Food dyes cause testicular damage *via* up-regulation of pro-inflammatory cytokines and down-regulation of FSH-R and TESK-1 gene expression. JBRA Assist. Reprod. 25 (3), 341–348. 10.5935/1518-0557.20200097 33565293 PMC8312291

[B48] Yadav H. M. Park J.-D. Kang H.-C. Lee J.-J. (2021). Recent development in nanomaterial-based electrochemical sensors for cholesterol detection. Chemosensors 9 (5), 98. 10.3390/chemosensors9050098

[B49] Yadav S. Jangra R. Sharma B. R. Sharma M. (2022). Current Advancement in biosensing techniques for determination of Alanine aminotransferase and Aspartate aminotransferase-a Mini Review. Process Biochem. 114, 71–76. 10.1016/j.procbio.2022.01.010

[B50] Yang S. J. Wang Y. S. Zhang L. D. Ding Z. M. Zhou X. Duan Z. Q. (2023). High-dose synthetic phenolic antioxidant propyl gallate impairs mouse oocyte meiotic maturation through inducing mitochondrial dysfunction and DNA damage. Environ. Toxicol. 38 (8), 1800–1810. 10.1002/tox.23807 37052413

[B51] Yasser H. Sabour A. N. (2023). Effects of the anthocyanin compound (Cyanidin-3-Glucoside) on some histological and physiological parameters related to the heart in Male rats exposed to oxidative stress. World's Veterinary J. 13 (1), 95–102. 10.54203/scil.2023.wvj10

[B52] Zhang J. Zuo B. Poklar Ulrih N. Sengupta P. K. Zheng X. Xiao J. (2018). Structure-affinity relationship of dietary anthocyanin–HSA interaction. J. Berry Res. 8 (1), 1–9. 10.3233/JBR-170167

